# The Electrical Conductivity of the Blood and Urine

**Published:** 1907-02-09

**Authors:** 


### THE ELECTRICAL CONDUCTIVITY OF THE BLOOD AND URINE.

Dr. Dawson Turner recently communicated to the Medico-Chirurgical Society of Edinburgh the results of a research on the electrical conductivity of the blood and urine in health and in disease and as a test of the functional efficiency of the kidney. The resistance of the urine was estimated in the usual manner by means of a U-tube. In health the resistance was low (about 250 ohms), being less as the proportion of salts present in the urine is higher—*i.e.* the more efficient the excretion by the kidney. In disease the electrical resistance of the urine was usually increased, but the cause of this increase was not always easily accounted for; thus in pneumonia, the high resistance obtained is due to the absence of chlorides; but in diabetes it does not depend on the presence of sugar, as artificial solutions of glucose of corresponding strength do not present a correspondingly high resistance.

The estimation of the resistance of the blood presented many difficulties, owing to the small quantities available; but by the use of an ingenious application of a micrometer screw with a bridge contact, Dr. Turner could obtain the resistance in quantities as small as five cubic millimetres. The normal resistance of the blood is high (about 900 ohms), the conductivity being impeded by the corpuscles; and any alteration in the number of these influences the resistance. The most marked alteration in disease is observed in pernicious anæmia, where the resistance falls in proportion to the blood-count.

Dr. Turner has recently combined the observed resistances of the blood and urine in the same patient in the form of a fraction by dividing the former by the latter, which gives a value in health of three and upwards. This ratio it had been proposed to call the "hæmo-renal salt index," and it seemed probable that it might afford valuable information as to the excretory power of the kidney, especially when combined with segregation of the urine in cases where nephrectomy was proposed. In cases where it was proposed to remove the kidney the urine of the better kidney should have a hæmo-renal index of about three; below that figure the prognosis was less favourable; and when below two it was serious. Observations by this method had been as yet few in number, but in general they agreed with those obtained by cryoscopy.

